# Crystal structure of 4-hy­droxy­pyridin-1-ium 3,5-di­carb­oxy­benzoate

**DOI:** 10.1107/S2056989015011780

**Published:** 2015-06-27

**Authors:** Selena L. Staun, Allen G. Oliver

**Affiliations:** a Department of Chemistry and Biochemistry, University of Notre Dame, Notre Dame, IN 46556-5670, USA

**Keywords:** crystal structure, 4-hy­droxy­pyridin-1-ium, 3,5-di­carb­oxy­benzoate, hydrogen bonding, cocrystal

## Abstract

The structure of the title salt, C_5_H_6_NO^+^·C_9_H_5_O_6_^−^, (**I**), shows that 4-hy­droxy­pyridine has abstracted an H atom from benzene-1,3,5-tri­carb­oxy­lic acid, yielding a pyridinium cation and carboxyl­ate anion. The two ions form an extensive three-dimensional hydrogen-bonded network throughout the crystal. The hydrogen bonds that comprise the core of the network are considered strong, with O—H⋯O and N—H⋯O donor-to-acceptor distances ranging from 2.533 (2) to 2.700 (2) Å. Packing is further enhanced by π-stacking of the cations and anions with like species [centroid–centroid distance = 3.6206 (13) Å].

## Abstract 2

A 1:1.4 molar equivalent of benzene-1,3,5-tri­carb­oxy­lic acid cocrystallized with 4-hy­droxy­pyridine yields the 4-hy­droxy­pyridin-1-ium 3,5-di­carb­oxy­benzoate salt.

## Chemical Context   

As a study in crystal engineering utilizing hydrogen bonding between disparate mol­ecules (Desiraju, 2003 ▸), we have been investigating the cocrystallization of various pyridine compounds with benzene carb­oxy­lic acids (Staun & Oliver, 2012 ▸). From previous work, 4-hy­droxy­pyridine undergoes hydrogen migration from the hy­droxy O to the pyridine N atom, yielding 4-pyridone (Tyl *et al.*, 2008 ▸). We were surprised to find that in the case of 4-hy­droxy­pyridin-1-ium 3,5-di­carb­oxy­benzoate, (**I**), an H atom is abstracted from one carb­oxy­lic acid group, yielding a pyridinium salt. This result allows for the hy­droxy O and pyridine N atom to both act as hydrogen-bond donors, rather than the donor/acceptor situation of the 4-pyridone species. These two mol­ecules have been incorporated as linker species in metal–organic frameworks (Guo *et al.*, 2011 ▸).
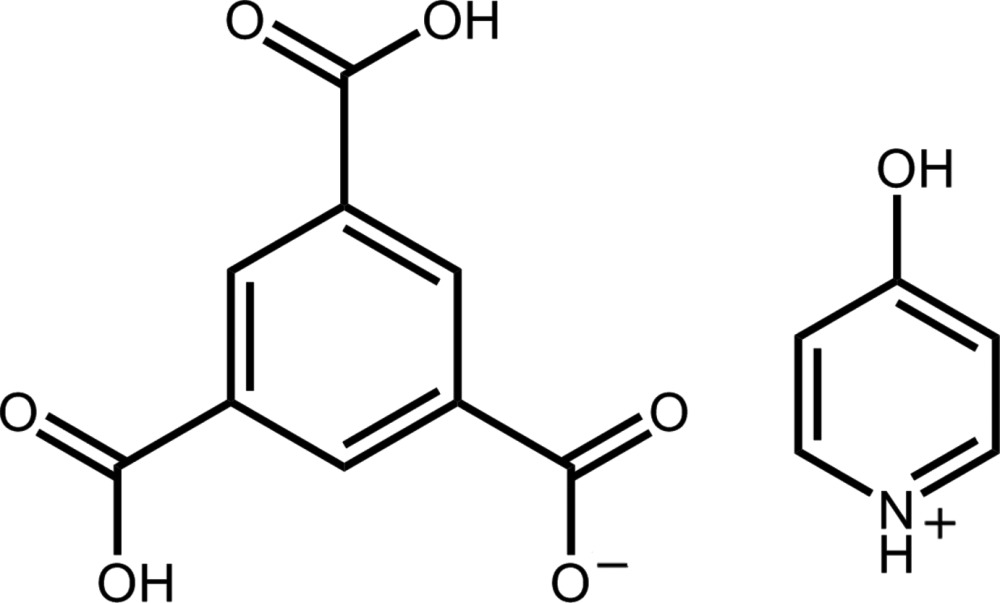


## Structural Commentary   

**Figure 1:**
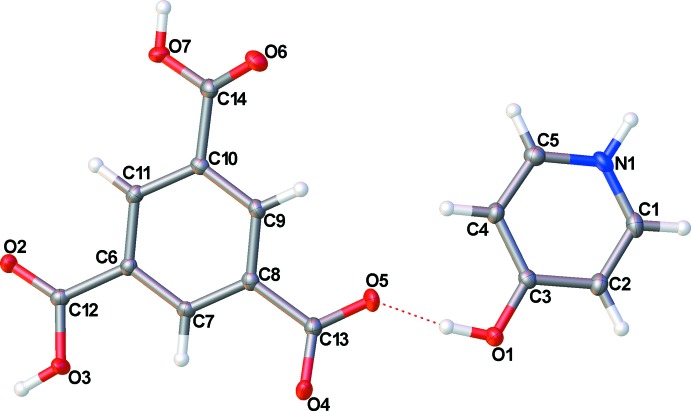
Labeling scheme for (**I**). Displacement ellipsoids are depicted at the 50% probability level. The inter-ion hydrogen bond is shown as a dashed red line.

The structure of (**I**) shows that the 4-hy­droxy­pyridine has abstracted an H atom from the benzene­tri­carb­oxy­lic acid, yielding a pyridinium cation and a carboxyl­ate anion (Fig. 1 ▸). Bond distances about the pyridine ring show some localization of the bonds: C1—C2 and C4—C5 are slightly shorter than the ideal aromatic distance [1.367 (3) and 1.369 (3) Å, respectively, *cf.* 1.390 Å for an aromatic C—C bond]. The N1—C1 and N1—C5 distances are typical for an aromatic N atom [1.345 (3) and 1.348 (3) Å, respectively]. The remaining bonds within the ring display typical aromatic distances [C2—C3 = 1.405 (3) Å and C3—C4 = 1.402 (3) Å]. The C3—O1 distance of 1.326 (2) Å is typical for a hy­droxy O atom bound to an aromatic ring. Bond angles within the pyridine ring are unexceptional.

Two of the three carb­oxy­lic acid groups show distinct single- and double-bond character [C12—O3 = 1.305 (3) Å and C14—O7 = 1.332 (3) Å; C12—O2 = 1.224 (2) Å and C14—O6 = 1.204 (3) Å]. The remaining carboxyl­ate group displays C—O bond distances that are similar to each other and indicate delocalization of the C—O bonds [1.268 (3) and 1.249 (2) Å for C13—O4 and C4—O5, respectively], supporting the proposed single negative charge on the benzene­tri­carb­oxy­lic acid mol­ecule. This is further supported by the presence of H atoms, located in a difference Fourier map, on atoms O3 and O7. Bond distances and angles within the benzene ring are as expected.

## Supra­molecular Features   

The local inter­molecular contacts consist of the pyridinium cation forming a hydrogen bond from the hy­droxy group to the anionic carboxyl­ate group (O1⋯O5; see Table 1 ▸ for detailed contacts) and from pyridine atom N1 to carboxyl­ate atom O4^i^ [symmetry code: (i) −*x* + 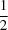, *y* + 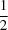, *z* − 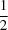]. Carb­oxy­lic acid atoms O3 and O7 are donors for hydrogen bonds to atoms O4^ii^ and O2^iii^, respectively [symmetry codes: (ii) −*x*, −*y*, *z* − 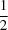; (iii) −*x*, −*y* + 1, *z* + 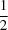]. Since these hydrogen bonds extend over several mol­ecules, an extensive hydrogen-bonded network exists in this structure.

**Figure 2:**
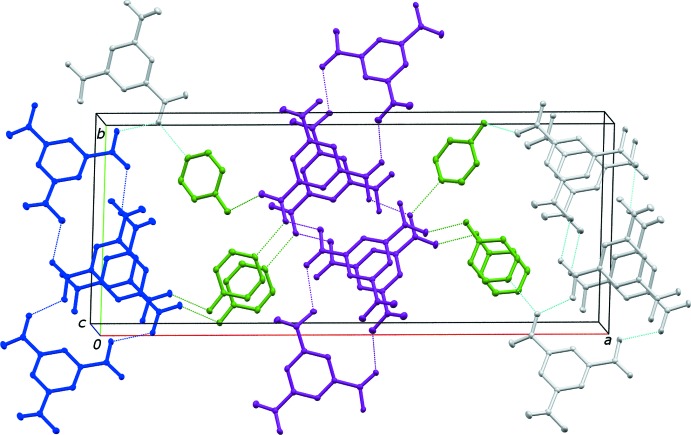
A view of (**I**) approximately along the crystallographic *c* axis. Color code: blue represents the 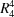
(28) ring, purple the 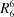
(44) ring, and green the bridging 4-hy­droxy­pyridinium cations.

Pertinent features of this extended network are an 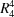
(28) ring comprised of 3,5-di­carb­oxy­benzoate ions (Fig. 2 ▸) (Bernstein *et al.*, 1995 ▸). The carb­oxy­lic acid groups are involved in the hydrogen bonding within this ring. There is also an 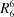
(44) ring of 3,5-di­carb­oxy­benzoate ions, that incorporate a different chain of carb­oxy­lic acid groups. These rings are bridged by the 4-hy­droxy­pyridinium cations resulting in the three-dimensional network. The hydrogen bonds within the structure are surprisingly strong, with O—H⋯O and N—H⋯O distances ranging from 2.533 (2) to 2.700 (2) Å (Table 1 ▸).

The cations and anions form homogeneous π-stacked columns parallel to the *c* axis, that is, 4-pyridinium cations stacking with other cations and 3,5-di­carb­oxy­benzoate anions stacking with other anions. The centroid-to-centroid distances for both the pyridinium and the di­carb­oxy­benzoate inter­actions are 3.6206 (13) Å, *i.e.* the *c*-axis spacing. The centroid-to-perpendicular distances are 3.3629 (9) Å for the cation and 3.4372 (9) Å for the anion. Both measurements are within accepted π–π contact ranges (see Table 2 ▸; Spek, 2009 ▸).

## Database Survey   

A search of the Cambridge Structural Database (CSD, Version 5.36 plus 3 updates; Groom & Allen, 2014 ▸) for 4-hy­droxy­pyridine and benzene­tri­carb­oxy­lic acid gave only five hits. In the compound that is most closely related to the title compound, namely benzene-1,3,5-tri­carb­oxy­lic acid pyridin-4(1*H*)-one (Campos-Gaxiola *et al.*, 2014 ▸), there are three mol­ecules of 4-pyridone present in the asymmetric unit. Benzene­tri­carb­oxy­lic acid and a tetra­kis­[(pyridin-4-yl­oxy)meth­yl]methane moiety (incorporating a 4-hy­droxy­pyridine functionality) have been utilized in the devlopment of frameworks incorporating copper and cadmium (Guo *et al.*, 2011 ▸).

## Synthesis and Crystallization   

To a solution of benzene-1,3,5-tri­carb­oxy­lic acid (0.035 g, 1.24 mmol) in MeOH (3 ml) in a 20 ml vial was added a solution of 4-hy­droxy­pyridine (0.0218 g, 1.77 mmol) in MeOH (3 ml). The mixture was shaken vigorously, covered with perforated Parafilm and allowed to evaporate slowly over a period of 5 d, yielding colorless rod-like crystals.

## Refinement   

Crystal data, data collection and structure refinement details are summarized in Table 3 ▸. Carb­oxy­lic, hy­droxy, and pyridinium H atoms were initally located in a difference Fourier map. H atoms on the 4-hy­droxy­pyridinium cation were refined freely. H atoms on the carb­oxy­lic acid groups were included with refined coordinates and atomic displacement parameters tied to that of the O atom to which they are bonded. C—H hydrogens were included in idealized positions riding on the C atom to which they are bonded, with C—H distances constrained to 0.95 Å and *U*_iso_(H) = 1.2 *U*_eq_(C).

The compound is achiral, but crystallizes with a noncentrosymmetric, polar space group. The Flack *x* parameter refined to 0.20 (8), which suggests the possibility of a small amount of inversion twinnning (Parsons *et al.*, 2013 ▸), but the strength of the anomalous signal is very weak. We compared both a model twinned by inversion and the untwinned model, and there was no significant difference. We therefore elected to model the structure without inclusion of a twin component.

## Supplementary Material

Crystal structure: contains datablock(s) I, New_Global_Publ_Block. DOI: 10.1107/S2056989015011780/pk2555sup1.cif

Structure factors: contains datablock(s) I. DOI: 10.1107/S2056989015011780/pk2555Isup2.hkl

Supporting information file. DOI: 10.1107/S2056989015011780/pk2555Isup3.cml

CCDC reference: 1407819

Additional supporting information:  crystallographic information; 3D view; checkCIF report

## Figures and Tables

**Table 1:** Hydrogen-bond geometry (, )

*D*H*A*	*D*H	H*A*	*D* *A*	*D*H*A*
O1H1OO5	0.95(3)	1.59(4)	2.533(2)	171(3)
N1H1NO4^i^	0.98(3)	1.73(3)	2.700(2)	173(4)
O3H3OO4^ii^	0.93(3)	1.65(3)	2.574(2)	172(3)
O7H7OO2^iii^	0.90(3)	1.79(3)	2.678(2)	166(3)

Symmetry codes: (i) 
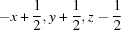
; (ii) 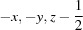; (iii) 
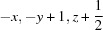
.

**Table 2:** -stacking interactions within (I)

Interaction	*Cg* *Cg* ()	*Cg*perp ()
*Cg*1*Cg*1^i^	3.6206(13)	3.4373(9)
*Cg*2*Cg*2^i^	3.6206(13)	3.3627(9)

*Cg*1 is the centroid of the 3,5-dicarboxybenzoate ring, *Cg*2 is the centroid of the 4-hydroxypyridinium ring [symmetry code: (i) *x*, *y*, 1+*z*], *Cg* *Cg* is the centroid-to-centroid distance, and *Cg*perp is the distance to the plane perpendicular to the ring centroid.

**Table 3:** Experimental details

Crystal data	
Chemical formula	C_5_H_6_NO^+^C_9_H_5_O_6_
*M*_r_	305.24
Crystal system, space group	Orthorhombic, *P* *n* *a*2_1_
Temperature (K)	122
*a*, *b*, *c* ()	29.3465(10), 12.2113(5), 3.6206(2)
*V* (^3^)	1297.47(10)
*Z*	4
Radiation type	Cu *K*
(mm^1^)	1.10
Crystal size (mm)	0.11 0.06 0.06

Data collection	
Diffractometer	Bruker APEXII
Absorption correction	Numerical (*SADABS*; Krause *et al.*, 2015 ▸)
*T*_min_, *T*_max_	0.694, 0.753
No. of measured, independent and observed [*I* > 2(*I*)] reflections	6000, 2322, 2172
*R*_int_	0.018
(sin /)_max_ (^1^)	0.614

Refinement	
*R*[*F* ^2^ > 2(*F* ^2^)], *wR*(*F* ^2^), *S*	0.028, 0.071, 1.05
No. of reflections	2322
No. of parameters	213
No. of restraints	1
H-atom treatment	H atoms treated by a mixture of independent and constrained refinement
_max_, _min_ (e ^3^)	0.15, 0.19
Absolute structure	Flack *x* determined using 786 quotients [(*I* ^+^)(*I* )]/[(*I* ^+^)+(*I* )] (Parsons *et al.*, 2013 ▸)
Absolute structure parameter	0.20(8)

Computer programs: *APEX2* and *SAINT* (Bruker 2012 ▸), *SHELXS97* (Sheldrick, 2008 ▸), *SHELXL2014* (Sheldrick, 2015 ▸), *OLEX2* (Dolomanov *et al.*, 2009 ▸), *Mercury* (Macrae *et al.*, 2008 ▸), *POVRay* (Cason, 2003 ▸), *publCIF* (Westrip, 2010 ▸) and *PLATON* (Spek, 2009 ▸).

## supplementary crystallographic information

### Crystal data

**Table:**

C_5_H_6_NO^+^·C_9_H_5_O_6_^−^	*D*_x_ = 1.563 Mg m^−^^3^
*M**_r_* = 305.24	Cu *K*α radiation, λ = 1.54184 Å
Orthorhombic, *P**n**a*2_1_	Cell parameters from 2373 reflections
*a* = 29.3465 (10) Å	θ = 3.4–71.2°
*b* = 12.2113 (5) Å	µ = 1.10 mm^−^^1^
*c* = 3.6206 (2) Å	*T* = 122 K
*V* = 1297.47 (10) Å^3^	Rod, colorless
*Z* = 4	0.11 × 0.06 × 0.06 mm
*F*(000) = 632	

### Data collection

**Table:**

Bruker APEXII diffractometer	2322 independent reflections
Radiation source: Incoatec micro-focus	2172 reflections with *I* > 2σ(*I*)
Detector resolution: 8.33 pixels mm^-1^	*R*_int_ = 0.018
combination of ω and φ–scans	θ_max_ = 71.2°, θ_min_ = 3.0°
Absorption correction: numerical *SADABS* (Krause *et al.*, 2015)	*h* = −27→35
*T*_min_ = 0.694, *T*_max_ = 0.753	*k* = −13→14
6000 measured reflections	*l* = −4→4

### Refinement

**Table:**

Refinement on *F*^2^	Secondary atom site location: difference Fourier map
Least-squares matrix: full	Hydrogen site location: mixed
*R*[*F*^2^ > 2σ(*F*^2^)] = 0.028	H atoms treated by a mixture of independent and constrained refinement
*wR*(*F*^2^) = 0.071	*w* = 1/[σ^2^(*F*_o_^2^) + (0.0441*P*)^2^ + 0.1815*P*] where *P* = (*F*_o_^2^ + 2*F*_c_^2^)/3
*S* = 1.05	(Δ/σ)_max_ = 0.001
2322 reflections	Δρ_max_ = 0.15 e Å^−^^3^
213 parameters	Δρ_min_ = −0.19 e Å^−^^3^
1 restraint	Absolute structure: Flack *x* determined using 786 quotients [(I+)-(I-)]/[(I+)+(I-)] (Parsons *et al.*, 2013)
Primary atom site location: structure-invariant direct methods	Absolute structure parameter: 0.20 (8)

### Special details

**Table:**

Geometry. All e.s.d.'s (except the e.s.d. in the dihedral angle between two l.s. planes) are estimated using the full covariance matrix. The cell e.s.d.'s are taken into account individually in the estimation of e.s.d.'s in distances, angles and torsion angles; correlations between e.s.d.'s in cell parameters are only used when they are defined by crystal symmetry. An approximate (isotropic) treatment of cell e.s.d.'s is used for estimating e.s.d.'s involving l.s. planes.

### Fractional atomic coordinates and isotropic or equivalent isotropic displacement parameters (Å^2^)

**Table:**

	*x*	*y*	*z*	*U*_iso_*/*U*_eq_	
O1	0.23997 (5)	0.04165 (12)	0.3072 (5)	0.0195 (4)	
H1O	0.2112 (11)	0.062 (3)	0.405 (11)	0.057 (11)*	
N1	0.32385 (6)	0.30371 (16)	0.1767 (5)	0.0181 (4)	
H1N	0.3450 (9)	0.365 (3)	0.166 (11)	0.048 (10)*	
C1	0.33796 (7)	0.2029 (2)	0.0796 (6)	0.0186 (5)	
H1A	0.3679	0.1933	−0.0157	0.022*	
C2	0.30997 (6)	0.11410 (19)	0.1159 (7)	0.0169 (4)	
H2A	0.3201	0.0433	0.0444	0.020*	
C3	0.26602 (6)	0.12932 (17)	0.2608 (6)	0.0145 (4)	
C4	0.25177 (7)	0.23551 (18)	0.3521 (6)	0.0160 (4)	
H4A	0.2218	0.2481	0.4424	0.019*	
C5	0.28155 (7)	0.32085 (18)	0.3095 (6)	0.0174 (4)	
H5A	0.2723	0.3929	0.3741	0.021*	
O2	−0.06609 (4)	0.25817 (12)	0.5787 (5)	0.0182 (3)	
O3	−0.04180 (5)	0.08670 (13)	0.5070 (5)	0.0215 (4)	
H3O	−0.0703 (9)	0.067 (2)	0.417 (9)	0.032*	
O4	0.11707 (4)	−0.02696 (12)	0.7027 (5)	0.0196 (4)	
O5	0.16718 (4)	0.10740 (13)	0.6111 (6)	0.0252 (4)	
O6	0.11934 (5)	0.47723 (13)	1.1519 (5)	0.0220 (4)	
O7	0.05337 (5)	0.53129 (13)	0.8992 (5)	0.0204 (4)	
H7O	0.0611 (8)	0.598 (3)	0.987 (9)	0.031*	
C6	0.01288 (6)	0.21569 (17)	0.6846 (6)	0.0127 (4)	
C7	0.04618 (7)	0.13490 (17)	0.6462 (6)	0.0128 (4)	
H7A	0.0380	0.0638	0.5628	0.015*	
C8	0.09153 (6)	0.15874 (17)	0.7304 (6)	0.0133 (4)	
C9	0.10330 (6)	0.26321 (18)	0.8480 (6)	0.0142 (4)	
H9A	0.1341	0.2795	0.9056	0.017*	
C10	0.07019 (6)	0.34410 (18)	0.8820 (6)	0.0131 (4)	
C11	0.02463 (6)	0.32021 (17)	0.8012 (6)	0.0134 (4)	
H11A	0.0019	0.3751	0.8261	0.016*	
C12	−0.03557 (6)	0.18988 (17)	0.5860 (6)	0.0138 (4)	
C13	0.12794 (6)	0.07323 (18)	0.6782 (6)	0.0150 (4)	
C14	0.08419 (7)	0.45642 (18)	0.9953 (6)	0.0156 (5)	

### Atomic displacement parameters (Å^2^)

**Table:**

	*U*^11^	*U*^22^	*U*^33^	*U*^12^	*U*^13^	*U*^23^
O1	0.0143 (7)	0.0151 (8)	0.0291 (9)	−0.0021 (6)	0.0044 (7)	−0.0012 (7)
N1	0.0152 (8)	0.0205 (10)	0.0185 (9)	−0.0067 (8)	−0.0004 (7)	0.0002 (8)
C1	0.0122 (9)	0.0270 (12)	0.0166 (11)	−0.0006 (9)	0.0006 (8)	0.0004 (10)
C2	0.0142 (9)	0.0208 (11)	0.0157 (10)	0.0018 (8)	0.0008 (8)	−0.0017 (9)
C3	0.0133 (9)	0.0166 (11)	0.0135 (10)	−0.0014 (8)	−0.0015 (8)	−0.0005 (9)
C4	0.0132 (9)	0.0180 (11)	0.0170 (10)	0.0010 (8)	0.0016 (8)	−0.0006 (10)
C5	0.0190 (10)	0.0171 (11)	0.0161 (11)	−0.0012 (8)	0.0000 (8)	−0.0007 (9)
O2	0.0116 (6)	0.0130 (7)	0.0299 (9)	0.0009 (5)	−0.0012 (6)	0.0000 (7)
O3	0.0124 (7)	0.0138 (8)	0.0382 (11)	−0.0007 (6)	−0.0073 (7)	−0.0059 (7)
O4	0.0122 (6)	0.0134 (7)	0.0331 (9)	0.0017 (6)	0.0034 (6)	0.0022 (7)
O5	0.0120 (7)	0.0220 (9)	0.0417 (11)	−0.0023 (6)	0.0085 (7)	−0.0032 (8)
O6	0.0179 (7)	0.0204 (8)	0.0277 (9)	−0.0050 (6)	−0.0047 (7)	−0.0032 (7)
O7	0.0179 (8)	0.0126 (8)	0.0308 (10)	0.0003 (6)	−0.0019 (7)	−0.0057 (7)
C6	0.0125 (8)	0.0126 (10)	0.0130 (9)	0.0008 (8)	0.0013 (7)	0.0010 (8)
C7	0.0142 (8)	0.0112 (10)	0.0129 (10)	−0.0014 (7)	0.0007 (7)	0.0018 (8)
C8	0.0128 (9)	0.0143 (10)	0.0129 (10)	−0.0005 (8)	0.0009 (8)	0.0022 (9)
C9	0.0111 (9)	0.0166 (11)	0.0150 (10)	−0.0017 (8)	−0.0008 (8)	0.0002 (9)
C10	0.0139 (9)	0.0136 (10)	0.0117 (10)	−0.0017 (8)	0.0003 (8)	0.0002 (8)
C11	0.0139 (9)	0.0138 (10)	0.0127 (10)	0.0024 (8)	0.0004 (8)	0.0006 (8)
C12	0.0152 (9)	0.0125 (10)	0.0137 (10)	0.0000 (8)	−0.0002 (8)	0.0009 (9)
C13	0.0129 (9)	0.0159 (11)	0.0164 (10)	0.0001 (8)	0.0000 (8)	−0.0007 (9)
C14	0.0139 (9)	0.0168 (11)	0.0162 (11)	−0.0012 (8)	0.0026 (9)	−0.0015 (9)

### Geometric parameters (Å, º)

**Table:**

O1—C3	1.326 (2)	O5—C13	1.249 (2)
O1—H1O	0.95 (3)	O6—C14	1.204 (3)
N1—C1	1.345 (3)	O7—C14	1.332 (3)
N1—C5	1.348 (3)	O7—H7O	0.90 (3)
N1—H1N	0.98 (3)	C6—C11	1.388 (3)
C1—C2	1.367 (3)	C6—C7	1.396 (3)
C1—H1A	0.9500	C6—C12	1.499 (3)
C2—C3	1.405 (3)	C7—C8	1.396 (3)
C2—H2A	0.9500	C7—H7A	0.9500
C3—C4	1.402 (3)	C8—C9	1.389 (3)
C4—C5	1.369 (3)	C8—C13	1.506 (3)
C4—H4A	0.9500	C9—C10	1.391 (3)
C5—H5A	0.9500	C9—H9A	0.9500
O2—C12	1.224 (2)	C10—C11	1.399 (3)
O3—C12	1.305 (3)	C10—C14	1.489 (3)
O3—H3O	0.93 (3)	C11—H11A	0.9500
O4—C13	1.268 (3)		
			
C3—O1—H1O	111 (2)	C6—C7—C8	119.90 (19)
C1—N1—C5	121.26 (19)	C6—C7—H7A	120.1
C1—N1—H1N	119.9 (18)	C8—C7—H7A	120.1
C5—N1—H1N	118.7 (18)	C9—C8—C7	119.70 (18)
N1—C1—C2	121.04 (19)	C9—C8—C13	119.95 (17)
N1—C1—H1A	119.5	C7—C8—C13	120.29 (19)
C2—C1—H1A	119.5	C8—C9—C10	120.39 (18)
C1—C2—C3	118.9 (2)	C8—C9—H9A	119.8
C1—C2—H2A	120.6	C10—C9—H9A	119.8
C3—C2—H2A	120.6	C9—C10—C11	120.1 (2)
O1—C3—C4	123.01 (18)	C9—C10—C14	119.06 (17)
O1—C3—C2	118.02 (19)	C11—C10—C14	120.86 (18)
C4—C3—C2	118.97 (19)	C6—C11—C10	119.51 (18)
C5—C4—C3	119.15 (19)	C6—C11—H11A	120.2
C5—C4—H4A	120.4	C10—C11—H11A	120.2
C3—C4—H4A	120.4	O2—C12—O3	123.40 (18)
N1—C5—C4	120.7 (2)	O2—C12—C6	123.77 (19)
N1—C5—H5A	119.7	O3—C12—C6	112.83 (17)
C4—C5—H5A	119.7	O5—C13—O4	124.62 (19)
C12—O3—H3O	117.1 (18)	O5—C13—C8	116.56 (19)
C14—O7—H7O	110.8 (17)	O4—C13—C8	118.82 (17)
C11—C6—C7	120.42 (17)	O6—C14—O7	124.0 (2)
C11—C6—C12	120.09 (17)	O6—C14—C10	124.07 (19)
C7—C6—C12	119.45 (18)	O7—C14—C10	111.89 (17)
			
C5—N1—C1—C2	−0.7 (3)	C7—C6—C11—C10	−0.3 (3)
N1—C1—C2—C3	−0.7 (3)	C12—C6—C11—C10	−178.11 (19)
C1—C2—C3—O1	−177.5 (2)	C9—C10—C11—C6	−0.5 (3)
C1—C2—C3—C4	2.1 (3)	C14—C10—C11—C6	177.9 (2)
O1—C3—C4—C5	177.3 (2)	C11—C6—C12—O2	4.8 (4)
C2—C3—C4—C5	−2.2 (3)	C7—C6—C12—O2	−173.0 (2)
C1—N1—C5—C4	0.5 (3)	C11—C6—C12—O3	−175.4 (2)
C3—C4—C5—N1	0.9 (3)	C7—C6—C12—O3	6.7 (3)
C11—C6—C7—C8	0.9 (3)	C9—C8—C13—O5	−25.5 (3)
C12—C6—C7—C8	178.7 (2)	C7—C8—C13—O5	151.8 (2)
C6—C7—C8—C9	−0.7 (3)	C9—C8—C13—O4	154.9 (2)
C6—C7—C8—C13	−178.04 (19)	C7—C8—C13—O4	−27.8 (3)
C7—C8—C9—C10	−0.1 (3)	C9—C10—C14—O6	−20.3 (3)
C13—C8—C9—C10	177.3 (2)	C11—C10—C14—O6	161.3 (2)
C8—C9—C10—C11	0.7 (3)	C9—C10—C14—O7	159.0 (2)
C8—C9—C10—C14	−177.74 (19)	C11—C10—C14—O7	−19.4 (3)

### Hydrogen-bond geometry (Å, º)

**Table:**

*D*—H···*A*	*D*—H	H···*A*	*D*···*A*	*D*—H···*A*
O1—H1*O*···O5	0.95 (3)	1.59 (4)	2.533 (2)	171 (3)
N1—H1*N*···O4^i^	0.98 (3)	1.73 (3)	2.700 (2)	173 (4)
O3—H3*O*···O4^ii^	0.93 (3)	1.65 (3)	2.574 (2)	172 (3)
O7—H7*O*···O2^iii^	0.90 (3)	1.79 (3)	2.678 (2)	166 (3)

Symmetry codes: (i) −*x*+1/2, *y*+1/2, *z*−1/2; (ii) −*x*, −*y*, *z*−1/2; (iii) −*x*, −*y*+1, *z*+1/2.

## References

[bb1] Bernstein, J., Davis, R. E., Shimoni, L. & Chang, N.-L. (1995). *Angew. Chem. Int. Ed. Engl.* **34**, 1555–1573.

[bb2] Bruker (2012). *APEX2* and *SAINT*. Bruker–Nonius AXS Inc. Madison, Wisconsin, USA.

[bb3] Campos-Gaxiola, J. J., Zamora Falcon, F., Corral Higuera, R., Höpfl, H. & Cruz-Enríquez, A. (2014). *Acta Cryst.* E**70**, o453–o454. 10.1107/S1600536814005534 PMC3998530 24826154

[bb4] Cason, C. J. (2003). *POV-RAY*. Persistence of Vision Ray Tracer Pty Ltd, Victoria, Australia.

[bb5] Desiraju, G. R. (2003). *J. Mol. Struct.* **656**, 5–15.

[bb6] Dolomanov, O. V., Bourhis, L. J., Gildea, R. J., Howard, J. A. K. & Puschmann, H. (2009). *J. Appl. Cryst.* **42**, 339–341.

[bb7] Groom, C. R. & Allen, F. H. (2014). *Angew. Chem. Int. Ed.* **53**, 662–671. 10.1002/anie.201306438 24382699

[bb8] Guo, J., Ma, J.-F., Liu, B., Kan, W.-Q. & Yang, J. (2011). *Cryst. Growth Des.* **11**, 3609–3621.

[bb9] Krause, L., Herbst-Irmer, R., Sheldrick, G. M. & Stalke, D. (2015). *J. Appl. Cryst.* **48**, 3–10. 10.1107/S1600576714022985 PMC4453166 26089746

[bb10] Macrae, C. F., Bruno, I. J., Chisholm, J. A., Edgington, P. R., McCabe, P., Pidcock, E., Rodriguez-Monge, L., Taylor, R., van de Streek, J. & Wood, P. A. (2008). *J. Appl. Cryst.* **41**, 466–470.

[bb11] Parsons, S., Flack, H. D. & Wagner, T. (2013). *Acta Cryst.* B**69**, 249–259. 10.1107/S2052519213010014 PMC3661305 23719469

[bb12] Sheldrick, G. M. (2008). *Acta Cryst.* A**64**, 112–122. 10.1107/S0108767307043930 18156677

[bb13] Sheldrick, G. M. (2015). *Acta Cryst.* C**71**, 3–8.

[bb14] Spek, A. L. (2009). *Acta Cryst.* D**65**, 148–155. 10.1107/S090744490804362X PMC2631630 19171970

[bb15] Staun, S. L. & Oliver, A. G. (2012). *Acta Cryst.* C**68**, o84–o87. 10.1107/S0108270111055260 22307259

[bb16] Tyl, A., Nowak, M. & Kusz, J. (2008). *Acta Cryst.* C**64**, o661–o664. 10.1107/S0108270108033660 19057080

[bb17] Westrip, S. P. (2010). *J. Appl. Cryst.* **43**, 920–925.

